# Multiple thymi and no thymic involution in naked mole rats?

**DOI:** 10.1186/s12979-021-00253-w

**Published:** 2021-11-02

**Authors:** Graham Pawelec

**Affiliations:** Department of Immunology, University of Tübingen, Tübingen, Germany, Health Sciences North Research Institute, Sudbury, Ontario Canada

The naked mole rat (NMR) is a social mammal remarkable for any number of characteristics, most fascinatingly its extreme longevity. Little is known about its immune system, although as a subterranean animal immunity against infectious disease would be expected to be less important than in surface-dwelling social animals. Nonetheless, it provides an intriguing model for interactions of immune ageing with organismal ageing, which is likely to be of great importance, at least in mice [1]. Age-associated changes to the bone marrow niche and hematopoietic stem cells affect the production of T cell progenitors in mice in a dramatic manner [2], but the universal mammalian phenomenon of progressive thymic involution further decreases the capacity of the individual to generate new naïve T cells at older ages. Hence there has been a great deal of interest in preventing or reversing thymic involution in order to maintain diversity of the naïve TCR repertoire, required for the recognition of novel pathogens and cancer antigens. Thus, the hypothesis that such “thymic rejuvenation” would therefore benefit older adults has gained much traction over the years. The corollary to this hypothesis is that species or individuals within species that retain more and better thymic function into later life will be less likely to become “immunosenescent”, which would otherwise have negative consequences particularly for defence against pathogens to which they were not previously exposed.

Emmrich et al. [3] from the group of Vera Gorbunova now provide some fascinating information on the status of the thymus in NMRs, showing that in animals up to 11 years of age (still only middle-age for this species, but the oldest in their colony), there is no sign of thymic involution. Not only that, but, remarkably, NMRs are blessed with supplementary ectopic cervical thymi in addition to the usual thymus found (presumably exclusively) in the thoracic compartment of other mammals. Indeed, in young NMRs, anatomically, these “extra” thymi were larger than the thoracic thymus. Flow cytometry and single cell mRNA sequencing revealed the presence of all T cell differentiation stages in both the thoracic and cervical thymi. Unlike in mice, where the thymus steadily involutes with increasing age, in NMRs the cervical thymi in particular increased in size. Moreover, the level of expression of the autoimmune regulator gene (AIRE), which is essential for negative T cell selection in the thymus and which is proportional to thymic involution in mice, did not change with age in the NMR, suggesting retention of function. Similarly, the level of expression of the important marker of thymic epithelial cell integrity FOXN1 was also retained in NMRs, unlike in mice. As the authors point out, thymic involution might still occur in later life, but the fact that unlike in mice and humans, thymic atrophy in not progressive in these NMRs mitigates against that possibility. The question remains as to the evolutionary reason not only for the lack of thymic involution, but the presence of extra thymi in NMRs?

The authors interpret their data in terms of thymic involution having a negative effect on immune defence due to a reduction in the naïve TCR repertoire with age, but despite the general acceptance of this thesis, there is surprisingly little evidence that this is indeed the case in humans [4] and recent results on SARS-CoV-2 vaccination in older adults questions whether the healthy elderly do have a deficit in their protective response to neoantigens [5]. Given the likely protected subterranean environment in which NMRs live with little pathogen challenge, one wonders whether retention of what is probably high-level T cell output serves some purpose other than pathogen defence. Here, of course, protection against cancer springs to mind. It is indeed the case that NMRs appear to be remarkably resistant to cancer, but the Gorbunova group has previously shown that there are reasons other than anti-cancer immunosurveillance that are probably primarily responsible for this. Nonetheless, this does not preclude the additional existence of heightened immune defence against cancer, but this has not yet been tested and would be difficult given the low or absent rate of cancer occurrence in these animals. There may also be other, mostly unanticipated or under-investigated reasons for maintaining T cell integrity over extended periods, for example in wound healing, neuroendocrine function and memory, etc. It will be very interesting to learn what will happen a decade from now when the oldest animals in the colony are not 11 years old (“equivalent” to humans in their 30’s) but have reached human “old age” > 60 years. The evolutionary and functional relationships between T cell immunity and organismal longevity across species continue to fascinate and to raise questions as to what may be relevant to human health and what may be adaptations to extreme environments which do not translate directly to the human situation.

## Data Availability

Not applicable.
